# Giant optical anisotropy in transition metal dichalcogenides for next-generation photonics

**DOI:** 10.1038/s41467-021-21139-x

**Published:** 2021-02-08

**Authors:** G. A. Ermolaev, D. V. Grudinin, Y. V. Stebunov, K. V. Voronin, V. G. Kravets, J. Duan, A. B. Mazitov, G. I. Tselikov, A. Bylinkin, D. I. Yakubovsky, S. M. Novikov, D. G. Baranov, A. Y. Nikitin, I. A. Kruglov, T. Shegai, P. Alonso-González, A. N. Grigorenko, A. V. Arsenin, K. S. Novoselov, V. S. Volkov

**Affiliations:** 1 Center for Photonics and 2D Materials, Moscow Institute of Physics and Technology, Dolgoprudny, Russia; 2 Skolkovo Institute of Science and Technology, Moscow, Russia; 3 National Graphene Institute (NGI), University of Manchester, Manchester, UK; 4 Department of Physics and Astronomy, University of Manchester, Manchester, UK; 5 Department of Physics, University of Oviedo, Oviedo, Spain; 6 Center of Research on Nanomaterials and Nanotechnology, CINN (CSIC-Universidad de Oviedo), El Entrego, Spain; 7 Dukhov Research Institute of Automatics (VNIIA), Moscow, Russia; 8 CIC nanoGUNE BRTA, Donostia-San Sebastián, Spain; 9 Department of Physics, Chalmers University of Technology, Göteborg, Sweden; 10 Donostia International Physics Center (DIPC), Donostia-San Sebastián, Spain; 11 IKERBASQUE, Basque Foundation for Science, Bilbao, Spain; 12 GrapheneTek, Skolkovo Innovation Center, Moscow, Russia; 13 Department of Materials Science and Engineering, National University of Singapore, Singapore, Singapore; 14 Chongqing 2D Materials Institute, Chongqing, China

**Keywords:** Nanophotonics and plasmonics, Metamaterials

## Abstract

Large optical anisotropy observed in a broad spectral range is of paramount importance for efficient light manipulation in countless devices. Although a giant anisotropy has been recently observed in the mid-infrared wavelength range, for visible and near-infrared spectral intervals, the problem remains acute with the highest reported birefringence values of 0.8 in BaTiS_3_ and h-BN crystals. This issue inspired an intensive search for giant optical anisotropy among natural and artificial materials. Here, we demonstrate that layered transition metal dichalcogenides (TMDCs) provide an answer to this quest owing to their fundamental differences between intralayer strong covalent bonding and weak interlayer van der Waals interaction. To do this, we made correlative far- and near-field characterizations validated by first-principle calculations that reveal a huge birefringence of 1.5 in the infrared and 3 in the visible light for MoS_2_. Our findings demonstrate that this remarkable anisotropy allows for tackling the diffraction limit enabling an avenue for on-chip next-generation photonics.

## Abstract (web summary)

Optical anisotropy in a broad spectral range is pivotal to efficient light manipulation. Here, the authors measure a birefringence of 1.5 in the infrared range and 3 in the visible light for MoS_2_.

## Introduction

Optical anisotropy plays a crucial role in light manipulation owing to birefringence phenomena, namely, doubling the incoming light into two different rays (called ordinary and extraordinary for uniaxial optical materials). It results in spatial and polarization separation^1^, through versatile optical components^2–4^, including polarizers, wave plates, multilayer mirrors, and phase-matching elements. The performance of these devices primarily depends on the phase retardance (*φ*) between ordinary and extraordinary rays, which is proportional to the thickness (*d*) of the device and the birefringence (Δ*n*) of the constituting materials. Thus, a large birefringence is very favorable and beneficial since it leads to more compact and efficient devices. Despite its great importance for high-performance optics, the currently used materials such as inorganic solids and liquid crystals possess a relatively small birefringence with typical values below 0.4^5–9^. Even the record-holders quasi-one-dimensional BaTiS_3_ and layered h-BN crystals improve this result by less than twofold (Δ*n* ~ 0.8)^10,11^. The problem is partially solved in the mid-infrared range by large anisotropy in the biaxial van der Waals (vdW) crystals α-MoO_3_ and α-V_2_O_5_^12,13^. Still, these materials become mostly isotropic in the visible and near-infrared light. Meanwhile, artificial design can offer large birefringence in metamaterials and metasurfaces^14^. However, its widespread usage is impeded by optical losses and fabrication challenges.

**Fig. 1:**
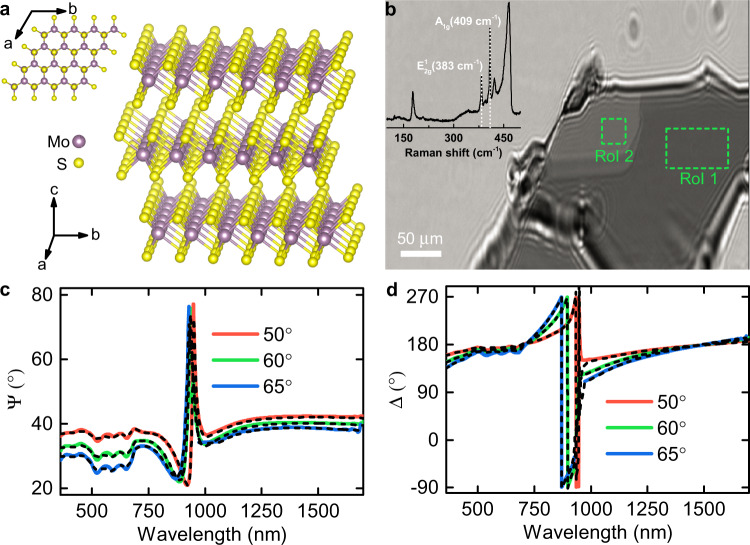
Anisotropy in MoS_2_. **a** Schematic illustration of the MoS_2_ layered structure: the giant anisotropy between *ab*-plane and c-axis arises from different interlayer (weak van der Waals bonding) and intralayer (strong ionic/covalent bonding) atomic interactions. **b** Optical ellipsometer microscope image of the exfoliated MoS_2_ thin film on a 285 nm-thick SiO_2_/Si substrate at 65°. The ellipsometry measurements were performed in two uniform areas marked by green dashed lines. The inset shows a resonant Raman spectrum at an excitation wavelength *λ* = 632.8 nm with the characteristic modes $E_{2g}^1$ = 383 cm^−1^ and *A*_1*g*_ = 409 cm^−1^, whose positions confirm the 2H semiconducting material configuration. **c**, **d** Experimental (solid lines) and analytically calculated (dashed lines, see Methods) ellipsometric parameters *Ψ* and *Δ* for ROI 1 (*Ψ* and *Δ* for ROI 2 see in Supplementary Note 2) at three incident angles 50°, 60°, and 65°. The asymmetric interference-like peak at around 900 nm is induced by interference enhancement in SiO_2_ caused by splitting the incident beam into ordinary and extraordinary beams indicating a giant anisotropy in MoS_2_.

As a result, natural materials with giant anisotropy (Δ*n* > 1) are in growing demand both for scientific and industrial purposes. In this regard, transition-metal dichalcogenides (TMDCs) in a bulk configuration are promising candidates because of their strongly anisotropic vdW structure, which naturally leads to a large intrinsic birefringence. In particular, while MoS_2_ solids adopt an in-plane crystalline layered structure through strong ionic/covalent bonds between molybdenum (Mo) and sulfur (S) atoms, the out-of-plane link of these layers occurs via weak vdW forces in trigonal prismatic configuration^15^, as illustrated in Fig. 1a.

As a consequence, a strong optical anisotropy emerges in TMDCs. A diagonal permittivity tensor can describe it with two optical constants corresponding to the crystallographic *ab*-plane and the *c*-axis^16^. Interestingly, these anisotropic properties of TMDCs were qualitatively demonstrated back in 1967 by Liang et al.^17^, but only currently attracted significant importance in experiments dealing with novel regimes of light-matter interactions^18,19^ comprising exciton-polariton transport^20^, Zenneck surface waves^21^, tunable modal birefringence^22^, and anapole-exciton polaritons^23^. Although a recent pioneering work by Hu et al.^16^ reported a birefringence value of Δ*n* = 1.4 for MoS_2_ at *λ* = 1530 nm, the values of asymmetric dielectric responses of MoS_2_ in a wide wavelength interval have so far remained unknown. It most likely stems from inherent experimental difficulties while measuring a high refractive index of anisotropic materials that we overcome here by joining together far and near-field characterization techniques. The method allows us to obtain the full dielectric tensor in a wide wavelength range (360–1700 nm) and reveals giant birefringence for MoS_2_ as high as Δ*n* ~ 1.5 in the infrared and Δ*n* ~  3 in the visible spectra. This outstandingly large optical anisotropy accompanied by a high refractive index *n* ~ 4 paves a way for highly efficient optics and light manipulation in photonic chips.

## Results

### Far- and near-field study of MoS_2_ anisotropic response

The measurement of the anisotropic optical response of TMDCs is a challenging task due to multiple experimental obstacles. One of the hardest parts is the implementation of traditional spectroscopic diffraction-limited characterization techniques, including transmittance, reflectance, and ellipsometry measurements, since bulk TMDCs are usually prepared by the exfoliation method, as a result, the samples obtained have lateral dimensions of tens of micrometers. A second difficulty is related to the measured signal’s low-sensitivity to the out-of-plane components attributed to a large in-plane refractive index *n* ~ 4. For instance, in the ellipsometry configuration, incident light at 80° gives the refraction angle of only 14.3°, according to the Snell’s law, which implies that the probed electric field of the refracted light mainly lies along the layers and thus, is almost insensitive to the out-of-plane dielectric component.

To overcome the latter, we prepared exfoliated thin MoS_2_ films on 285 nm SiO_2_/Si substrate and verified their 2H semiconducting configuration by the resonant Raman demonstrated in the inset of Fig. 1a since MoS_2_ exists in nature in three phase modifications (see details in Supplementary Note 1): semiconducting (2H and 3R) and metallic (1T)^24^. The thick layer of silicon oxide will produce an interference-like pattern for ordinary and extraordinary beams that can readily be detected employing phase-sensitive techniques such as spectroscopic ellipsometry. For this reason, we performed imaging spectroscopic ellipsometry (ISE) measurements in the 360–1700 nm wavelength range, given that it allows measuring samples down to several micrometers since it is a hybrid of ellipsometer and microscope (“Methods”). It allowed us to record the ellipsometry signal *Ψ* and *Δ* (“Methods”) from several regions of interest (ROI) of the flakes within the selected field of view, as indicated in Fig. 1b. As a result, multiple sample analysis was implemented to increase data reliability (see Supplementary Note 2). The resulting ellipsometry spectra in Fig. 1c show a pronounced asymmetrical interference-like peak at around 900 nm (see details in Supplementary Note 3). This is induced by a large phase difference between ordinary and extraordinary beams, indicating (without any modeling of the experimental curves) large birefringence stemming from a strong anisotropy between the *c*-axis and the *ab*-plane.

Notwithstanding the noticeable anisotropic feature at 900 nm in the measured spectra, to accurately retrieve the complete dielectric tensor of MoS_2_ and enable predictive capabilities for future advanced optical devices using this material, it is imperative to develop an accurate dielectric function model. In that case, the best route towards a dielectric description is to utilize the crystallographic features of MoS_2_. Briefly, in its 2H structure consecutive layers are linked by weak vdW forces and rotated by 180° with respect to each other leading to a strong suppression of interlayer hopping for both electrons and holes, and, thus, preventing the formation of tightly bound interlayer electron-hole pair upon light illumination, the so-called excitons^25,26^. Therefore, along the *c*-axis, the material is transparent, and a Cauchy model describes its dielectric response (see Supplementary Note 2), which is an evident consequence of the Kramers–Kronig relation between real (*n*) and imaginary (*k*) parts of the refractive index and material transparency. In contrast, the confinement of electrons and holes within the layer results in enormous binding energy (~50 meV) for intralayer A- and B- excitons at the visible range similar to its monolayer counterpart^27^. At the same time, it supports C and C’ exciton complexes at ultraviolet wavelengths due to the nest banding effects and complex atomic orbital contributions^28^. The Tauc-Lorentz oscillator model best describes this excitonic behavior for the *ab*-plane (see Supplementary Note 2) because it captures two of the most essential physical features:^29^ (i) at low photon energies, excitons cannot be excited, as a consequence, absorption, or equivalently the imaginary part of refractive index (*k*), is equal to zero in this wavelength range and (ii) excitonic peaks exhibit an asymmetric shape due to phonon coupling of bright (excited by light) and dark (not excited by light) excitons.

**Fig. 2:**
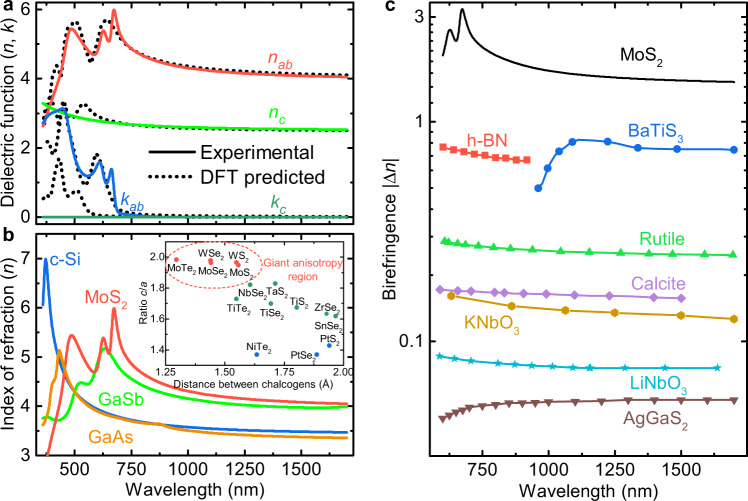
Optical anisotropy of MoS_2_. **a** Real (*n*) and imaginary (*k*) parts of the dielectric function along the *ab*-plane and *c*-axis. **b** Comparison of the MoS_2_ refractive index along the *ab*-plane with other high-refractive-index materials commonly used in nanophotonics^31–33^. The inset compares the ratio of the crystal parameters (*c* and *a* in Fig. 1a) versus the distance between the neighboring chalcogens (S, Se, and Te) for various TMDCs. The highest anisotropy is expected for TMDCs denoted by red circles. The crystallographic data were adopted from the review article^48^. **c** Comparison of the absolute birefringence values of MoS_2_ with different birefringent materials, including h-BN and BaTiS_3_, reported showing the highest anisotropy so far. The birefringence values for other materials in **c** were adopted from several reports^5–11^.

Using these models for describing the optical properties of MoS_2_, we fitted the experimentally measured ellipsometric parameters *Ψ* and *Δ* for both ROIs at the same time (see Supplementary Note 2). The resulting ordinary (along the layers) and extraordinary (perpendicular to the layers) optical constants and birefringence are displayed in Fig. 2a. These constants have a surprisingly well match with the values predicted by the first-principle calculations (see “Methods” and Supplementary Note 4). As expected, the material along the *c*-axis is transparent, even at ultraviolet and visible wavelengths. It confirms that excitons are formed in the layers providing a dichroic window from ~900 nm where the absorption of both ordinary and extraordinary light becomes negligible. Of immediate interest is the giant birefringence of Δ*n* ~ 1.5 in the infrared and Δ*n* ~ 3 in the visible ranges, which can serve as a platform for optical engineering in creating devices for the photonic application. As compared in Fig. 2c, the birefringence obtained for MoS_2_ in the visible and near-infrared spectral intervals is several times larger than for previous record-holders BaTiS_3_ and h-BN^10,11^, and an order of magnitude exceeding the values of currently used birefringent materials. Particular attention should also be given to the absolute values of the refractive indices, specifically their in-plane component. The high value of ~4.1 is comparable with traditionally used isotropic high-refractive-index semiconductors^30^, including Si (~3.6)^31^, GaAs (~3.5)^32^, and GaSb (~3.9)^33^ as illustrated in Fig. 2b. Such a large refractive index for MoS_2_ opens the door for lossless subwavelength photonics with the resolution of ~100 nm, which can easily rival with plasmonics platform, yet does not suffer from losses.

Furthermore, as we do not specify particular properties of MoS_2_ other than its in-plane excitonic nature and the out-of-plane transparency, our conclusions are quite general and can be applied to equally well to other semiconductor members of layered TMDCs with hexagonal, tetragonal, and trigonal atomic structure^1^. Consequently, we anticipate that other TMDCs with a hexagonal configuration also exhibit giant anisotropy because of the similarity in the crystal structure. In fact, the refractive index depends on the density of atoms in the crystal. As a consequence, one could expect that the larger the distance between the layers, the higher the birefringence value between in-plane and out-of-plane dielectric responses. Indeed, a comparison of their lattice parameters normalized to the distance between chalcogen intercore distances in the inset of Fig. 2b explains the giant anisotropy in MoS_2_ and forecasts similar (or even higher) birefringence values for MoSe_2_, WS_2_, WSe_2_, and MoTe_2_.

**Fig. 3:**
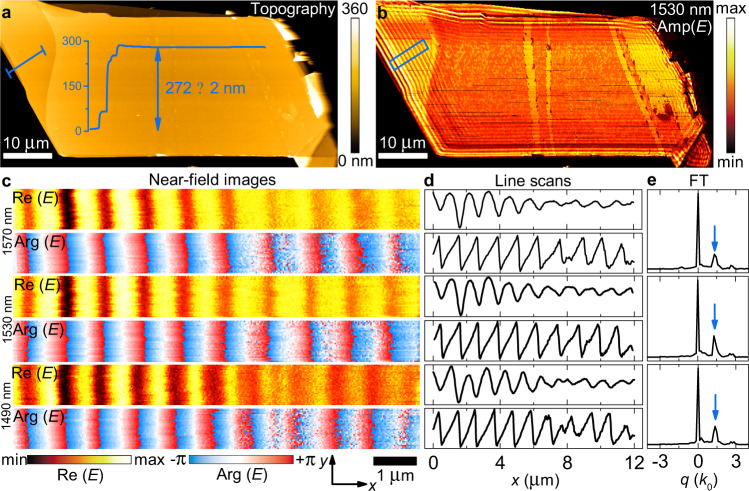
Waveguide modes imaged by s-SNOM. **a** Topography image of the analyzed MoS_2_ flake. A profile taken from the region labeled by blue line is shown. **b** Near-field amplitude image of the flake recorded at *λ* = 1530 nm. The region with the strongest signal is framed by a blue rectangular. **c** Near-field images, real part Re(E) and phase Arg(E), of the electric field E taken at 1570 nm (top), 1530 nm (middle), and 1490 nm (bottom) in the area of the image in **b**, indicated by a blue rectangle. **d** *x*-line scans taken from **c** and averaged over 1.2 μm along the *y*-axis (other wavelength images are collected in Supplementary Note 5). **e** Fourier transform (FT) amplitude of the complex near field signal in **d**. Blue arrows mark the peak associated with the waveguide mode.

**Fig. 4:**
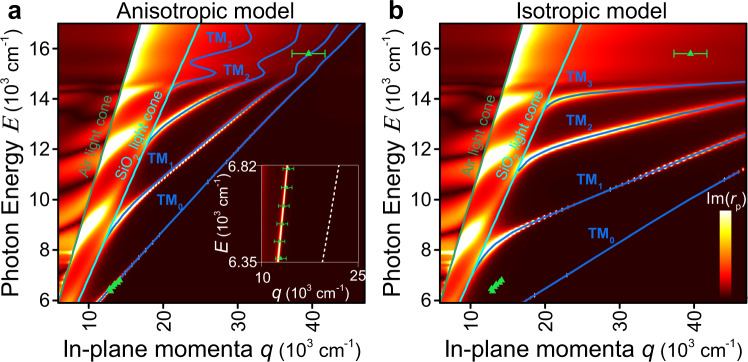
Dispersion of a planar MoS_2_ waveguide. **a**, **b** Transfer matrix calculations^49^ for the MoS_2_/SiO_2_/Si system for MoS_2_ anisotropic and isotropic (in the assumption of optical constants for the c-axis equal to those for the ab-plane) models. The experimental (*q* = 1/*λ*, *E* = *h*∙*c*/*λ*) data points (green triangles) show good agreement with the calculated dispersion (blue lines) based on the anisotropic dielectric function from Fig. 2a. The inset is a magnified near-infrared spectral range with a white dashed line illustrating the isotropic case’s dispersion relation. The error bars show the uncertainty in the determination of mode in-plane momenta *q* from near-field images displayed in Fig. 3.

For an unambiguous validation of the extracted dielectric function, we analyzed the planar transverse magnetic (TM) waveguide modes propagating in MoS_2_ flakes employing a scattering-type scanning near-field optical microscope (s-SNOM, “Methods”). By recording the scattered radiation, nanoscale images corresponding to the field distribution associated with the guided mode is obtained (Fig. 3a, b). The effective TM-waveguide mode index (*n*_eff,TM_) strongly depends on the material anisotropy allowing us to probe anisotropic response, in-plane (*n*_*ab*_) and out-of-plane (*n*_*c*_) refractive indices, and determined by^16^:

1 $$\frac{{2\pi d}}{\lambda }\sqrt {n_{ab}^2 - n_{{\mathrm{eff}},{\mathrm{TM}}}^2\frac{{n_{ab}^2}}{{n_c^2}}} = {\mathrm{tan}}^{ - 1}\left( {\frac{{n_{ab}^2}}{{n_{{\mathrm{Air}}}^2}}\frac{{\sqrt {n_{{\mathrm{eff}},{\mathrm{TM}}}^2 - n_{{\mathrm{Air}}}^2} }}{{\sqrt {n_{ab}^2 - n_{{\mathrm{eff}},{\mathrm{TM}}}^2\frac{{n_{ab}^2}}{{n_c^2}}} }}} \right) \\ +\, {\mathrm{tan}}^{ - 1}\left( {\frac{{n_{ab}^2}}{{n_{{\mathrm{SiO}}_2}^2}}\frac{{\sqrt {n_{{\mathrm{eff}},{\mathrm{TM}}}^2 - n_{{\mathrm{SiO}}_2}^2} }}{{\sqrt {n_{ab}^2 - n_{{\mathrm{eff}},{\mathrm{TM}}}^2\frac{{n_{ab}^2}}{{n_c^2}}} }}} \right) + m\pi ,$$

where *d* is the thickness of the MoS_2_ flake, *λ* is the incident wavelength of light, *n*_Air_ = 1 and $n_{{\mathrm{SiO}}_2}$ = 1.45 are the air and SiO_2_ refractive indices, and *m* is the mode order. We used incident wavelengths in the range 1470–1570 nm and 632.8 nm to excite guiding modes by focusing light into the apex of the s-SNOM tip, which allows for momentum matching conditions. The excited mode propagates in the MoS_2_ nanoflake as cylindrical waves, which interfere with the illuminating plane wave giving rise to interferometric patterns of the near-field^20^, as clearly seen in Fig. 3c, d. It is worth mentioning that while most of the previous works with s-SNOM for TMDCs focus only on the near-field amplitude^16,20^, the most accurate results are obtained by analyzing the phase as well^34^ (see Supplementary Note 5 for comparison). To retrieve the effective waveguide mode index, in Fig. 3e, we analyzed the Fourier transform (FT) of the individual line scans from Fig. 3d. The resulting FT has two pronounced peaks: one around zero due to background originating mostly from a strong tip-sample coupling. The second is associated with the planar TM-waveguide mode of interest. Note that there are no peaks in the left part of Fig. 3e (for negative values of *q*), indicating that no modes propagate in the backward direction (from the edge to the tip). The latter implies that mode scattering by the edge is far more efficient than the mode edge reflection or launching. Otherwise, we would observe standing waves with a cosine form, whose FT would be symmetrical and which are predominantly observed in nano-infrared imaging of graphene plasmons^35,36^ and hexagonal boron nitride (hBN) polaritons^37^. The primary reason for the observed tip-launching and edge-scattering mechanisms is the relatively small momenta of the modes^20^ since it is much closer to the free-space photon wavevector (*k*_0_) than in the studies of graphene^35,36^ or hBN^37^. For small momenta, the effective mode index is connected with that determined from the FT (*n*_s-SNOM,FT_) by momentum conservation along the edge direction^20^:

2 $$n_{{\mathrm{eff}},{\mathrm{TM}}} = n_{{\mathrm{s}} - {\mathrm{SNOM}},\,{\mathrm{FT}}} + {\mathrm{cos}}\left( \alpha \right) \cdot {\mathrm{sin}}\left( \beta \right)$$

where in our case $\alpha = 45^\circ$ is the angle between the illumination wavevector and its projection *k*_||_ on the sample surface plane and $\beta = 80^\circ$ is the angle between *k*_||_ and the sample edge. Given the extracted $n_{{\mathrm{eff}},{\mathrm{TM}}}$, we constructed the energy (*E* = *h∙c*/*λ*)–momentum (*q* = 1/*λ*) dispersion relation of the waveguide mode. The obtained experimental (*q, E*) data points (green triangles) are overlaid on top of the calculated dispersion color map in Fig. 4a using constants from Fig. 2a. For reference, we also added the dispersion for an isotropic model in Fig. 4b, assuming the optical constants to be the same for all crystallographic axes and equal one from the *ab*-plane. Notably, in the visible spectral range, where excitons start playing a role, the isotropic model (Fig. 4b) predicts the absence of guided modes owing to high material absorption. Conversely, our anisotropic results and near-field measurements reveal that even for this spectral interval, guided modes exist, which explains the recently discovered excitons polaritons in TMDCs^20^. Therefore, the excellent agreement between the experiment and theory validates our dielectric permittivity of MoS_2_, allowing for predicting capabilities in future photonic devices, including polarization-maintaining fibers^4^ and polarization tunable Mie-nanoresonators for nonlinear photonics^38^ since the magnetic dipole (MD) Mie-resonance in MoS_2_ nanoparticles is strongly affected by the refractive index values^39^. For instance, the spectral position of MD-resonance for a spherical particle is approximately defined by *λ*_MD_ ≈ *nD*, with *D* being the sphere’s diameter^40^. Besides, its anisotropic behavior allows its use as nanoresonators even for photon energies higher than the electronic bandgap thanks to the absence of absorption along the *c*-axis. At the same time, for conventional isotropic materials (c-Si, GaAs, and GaSb) this option is closed. Therefore, TMDCs provide device miniaturization, and their birefringence enables fine-tuning the resonance position in a wide spectral range by altering the light polarization, which is roughly *n*_*ab*_*D–n*_*c*_*D* ≈ 450 nm for a typical diameter of 300 nm.

### Extreme skin-depth waveguides based on giant anisotropy in MoS_2_

**Fig. 5:**
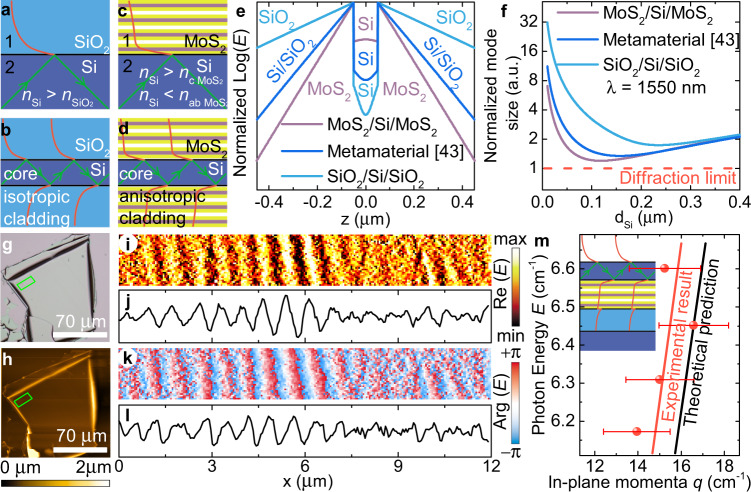
Transparent sub-diffraction optics. **a** Traditional total internal reflection with two isotropic media: above the critical angle, the light is reflected from the interface giving a decaying wave for a lower refractive index medium. **b** The decaying wave penetrates low refractive index material resulting in strong cross-talk between neighboring waveguides and limits the current on-chip photonics. **c** Relaxed total internal reflection: the out-of-plane dielectric refractive index is less than that of isotropic material, while the high in-plane refractive index provides fast decay of electric field amplitude in the first medium. **d** Anisotropic material gives rise to a planar waveguide with strong light confinement. The red curves for the **a**–**d** schematically show electric field amplitude. **e** Comparison of electric field distributions for MoS_2_/Si/MoS_2_, metamaterial(SiO_2_/Si)/Si/metamaterial(SiO_2_/Si)^43^ systems reveals that giant anisotropy causes giant light compression. **f** Light confinement in MoS_2_/Si/MoS_2_ allows for tackling the diffraction limit compared with traditionally used SiO_2_/Si/SiO_2_ and recently introduced^43^ metamaterial cladding with alternating layers of Si and SiO_2_ with silicon as a core. Mode size is normalized to the diffraction limit defined as *λ*/(2∙*n*_core_). **g**, **h** Optical and AFM topography images of the flake with 190 nm covered silicon. **i**–**l** Near field images, real part Re (*E*) and phase Arg (*E*), with the corresponding line scans of the electric field taken at *λ* = 1550 nm from the area of **g**–**h** indicated by a green rectangular. (s-SNOM images taken at different wavelengths are collected in Supplementary Note 5). **m** Comparison between theoretical and experimental mode dispersion. The error bars show the uncertainty in the determination of mode in-plane momenta *q* from near-field images displayed in **i**–**l**. The inset is an artistic representation of the investigated system Air/Si (195 nm)/MoS_2_ (285 nm)/SiO_2_ (285 nm)/Si.

More importantly, the giant anisotropy provides an avenue to resolve the fundamental limitations of isotropic materials. In this regard, the extreme skin-depth (e-skid) waveguide in Fig. 5 epitomizes the importance of a highly anisotropic material as an essential building block for next-generation photonics^41^. This phenomenon originates from the general law of total internal reflection (TIR). In the classical case, if *n*_2_ > *n*_1_, where *n*_1_ and *n*_2_ are the refractive indices of the medium 1 and 2 in Fig. 5a, and the incident angle is greater than the critical angle, light is reflected in the second medium and decays in the first. This effect is in the core of telecommunication technologies for guiding light at vast distances since light reflects without power loss. However, reducing the thickness of standard (typically Si) optical waveguides down to the nanometric scale introduces a considerable difficulty. As illustrated in Fig. 5a, b and e, f, the electromagnetic mode confinement in the optically less dense material significantly weakens. It restricts photonic integrated circuit miniaturization because of the cross-talk (electric field overlapping between adjacent waveguides)^42^. The solution to the problem is a generalized TIR form, which relaxes the condition *n*_2_ > *n*_1_ to *n*_2_ > *n*_c_ (Fig. 5c), where *n*_c_ is the out-of-plane refractive index of the first anisotropic medium^43^. Counterintuitively, the in-plane refractive index *n*_ab_ could have any value even higher than *n*_2_. More interestingly, the greater *n*_ab_, the better the light confinement inside the waveguide core (Fig. 5d), allowing us to get closer to the diffraction limit *λ*/2*n*_core_, where *λ* is the wavelength of light and *n*_core_ is the refractive index of the waveguide core. To experimentally demonstrate the effect, we covered a 285 nm-thick MoS_2_ flake with 190 nm-thick silicon (“Methods”) to form a planar e-skid waveguide Air/Si/MoS_2_. Although MoS_2_ is better than air for light confinement, we left one of the silicon slab faces uncovered to visualize the mode by near-field measurements shown in Fig. 5i–l. The waveguiding mode measured dispersion is in close agreement with the theoretical calculations, thus validating the e-skid waveguide concept for light confinement and, consequently, miniaturized photonic integrated circuits. It is worth mentioning that MoS_2_ yields markedly better light confinement than recently introduced metamaterial^41^ of alternating layers of Si and SiO_2_ (Fig. 5f) since MoS_2_ is a natural metamaterial with molybdenum, sulfur, and vdW gap varying layers (Fig. 5c, d). Finally, the proposed approach leads to another concept of a vertical integrated circuit, which has been recently proved to provide a useful degree of freedom for efficient light manipulation^44^.

## Discussion

Optical anisotropy lies behind the functionality of many optical devices such as polarizers and wave plates, to name a few. This phenomenon’s importance calls for active investigation of anisotropic materials to expand their application scope. However, the apparatus efficiency and compactness mostly depend on absolute birefringence values, which are moderate with the best result (Δ*n* < 0.8), reported in h-BN and BaTiS_3_ crystals in the visible and near-infrared ranges. We believe that these limitations can be outperformed by the family of TMDCs materials, whose inherent intralayer excitonic behavior results in large anisotropy along and perpendicular to the layers. To validate the concept, we showed a giant (Δ*n* > 1.5) broadband anisotropy for MoS_2_ employing far and near-field techniques. Additionally, we demonstrated its applicability for on-chip sub-duffraction optics. From a wider perspective, our result provides a path for next-generation nanophotonics based on TMDCs, for example, in tunable Mie-nanoresonators, and exciton-polariton physics.

## Methods

### Sample preparation

The MoS_2_ microcrystals were exfoliated on silicon wafers with 285-nm-thick thermal SiO_2_ from a synthetically grown bulk MoS_2_ sample purchased from the 2D Semiconductors Inc.

### Ellipsometry setup

ISE measurements were performed with a commercial spectroscopic nulling ellipsometer EP4 (https://accurion.com). Spectroscopic data are obtained in the spectral range 360–1700 nm in step with 1 nm. The light is guided through a polarizer for linear polarization and then through a compensator to prepare elliptically polarized collimated light so that the reflected light from the sample is again linearly polarized. The reflected light is directed through a 10× objective to a CCD camera (microscope configuration). In a suitable coordinate system, the complex reflectance matrix is described by tan(*Ψ*)∙exp(i*Δ*). The analytical *Ψ* and *Δ* are calculated using Fresnel formulas^1^.

### Near-field optical nano-spectroscopy

The nano-imaging recording was performed using a commercial s-SNOM (www.neaspec.com). The s-SNOM is based on a tapping-mode AFM illuminated by a monochromatic tunable laser of the wavelength from 1470–1570 nm spectral interval or He-Ne laser with the wavelength 632.8 nm. The near-field images were registered by pseudo-heterodyne interferometric module with tip-tapping frequency around 270 kHz with an amplitude of about 40 nm. The noise was significantly suppressed by demodulating the optical signal with a pseudo-heterodyne interferometer at high harmonics, *nΩ* (in our case third harmonics).

### Raman spectroscopy

The experimental setup used for Raman measurements was a confocal scanning Raman microscope Horiba LabRAM HR Evolution (https://www.horiba.com/). The measurements were carried out using linearly polarized excitation at a wavelengths of 532 and 632.8 nm, 300 lines/mm diffraction grating, and ×100 objective (N.A. = 0.90), whereas we used unpolarized detection to have a significant signal-to-noise ratio. The spot size was ~0.43 µm. The Raman spectra were recorded with 0.26 mW incident powers and an integration time of 10 s.

### First-principle calculations

Optical properties of 2H-MoS_2_ were calculated using density functional theory (DFT) within the generalized gradient approximation^44^ (Perdew–Burke–Ernzerhof functional) and the projector-augmented wave method^45^ as implemented in the Vienna Ab initio Simulation Package^46,47^. A two-step approach was used: first, MoS_2_ crystal structure was relaxed, and a one-electron basis set was obtained from a standard DFT calculation; second, micro- and macroscopic dielectric tensors were calculated using GW approximation. Plane wave kinetic energy cutoff was set to 400 eV, and the Г-centered k-points mesh sampled the Brillouin zone with a resolution of 2*π*∙0.05 Å^−1^.

## Supplementary information

Supplementary Information

## Data Availability

The datasets generated during and/or analyzed during the current study are available from the corresponding author on reasonable request.
