# Supplementary material for: Giant optical anisotropy in transition metal dichalcogenides for next-generation photonics
Source: Nat Commun. 2021 Feb 8;12:854. doi: 10.1038/s41467-021-21139-x (PMC7870936; doi:10.1038/s41467-021-21139-x)
Supplement: Supplementary file 1 — Supplementary Information [file 41467_2021_21139_MOESM1_ESM.pdf]

Supplementary Information

Giant optical anisotropy in transition metal dichalcogenides for the next-generation photonics

*G.A. Ermolaev[1,2], D.V. Grudinin[1], Y.V. Stebunov[3], K.V. Voronin[1,2], V.G. Kravets[4], J. Duan[5,6], A.B. Mazitov[1,7], G.I. Tselikov[1], A. Bylinkin[1,8], D.I. Yakubovsky[1], S.M. Novikov[1], D.G. Baranov[1,9], A.Y. Nikitin[10,11,1], I.A. Kruglov[1,7], T. Shegai[9], P. Alonso-González[5,6], A.N. Grigorenko[4], A.V. Arsenin[1,12], K.S. Novoselov[13,3,14], V.S. Volkov[1,12]**

[1]Center for Photonics and 2D Materials, Moscow Institute of Physics and Technology, Dolgoprudny 141700, Russia.

[2]Skolkovo Institute of Science and Technology, Moscow 121205, Russia.

[3]National Graphene Institute (NGI), University of Manchester, Manchester M13 9PL, UK.

[4]Department of Physics and Astronomy, University of Manchester, Manchester M13 9PL, UK.

[5]Department of Physics, University of Oviedo, Oviedo 33006, Spain.

[6]Center of Research on Nanomaterials and Nanotechnology, CINN (CSIC-Universidad de Oviedo), El Entrego 33940, Spain.

[7]Dukhov Research Institute of Automatics (VNIIA), Moscow 127055, Russia.

[8]CIC nanoGUNE BRTA, Donostia-San Sebastián 20018, Spain.

[9]Department of Physics, Chalmers University of Technology, Göteborg 412 96, Sweden.

[10]Donostia International Physics Center (DIPC), Donostia-San Sebastián 20018, Spain.

[11]IKERBASQUE, Basque Foundation for Science, Bilbao 48013, Spain.

[12]GrapheneTek, Skolkovo Innovation Center, Moscow 143026, Russia.

[13]Department of Materials Science and Engineering, National University of Singapore, Singapore 117574, Singapore.

[14]Chongqing 2D Materials Institute, Liangjiang New Area, Chongqing 400714, China.

*e-mail: volkov.vs@mipt.ru

Supplementary Note 1: Crystal characterization of 2H-MoS_2

Bulk MoS_2 is found to exist in three polymorphic states (different stacking sequences of monolayers with the same structure) shown in Supplementary Figure 1: 1T (tetragonal), 2H (hexagonal), and 3R (rhombohedral) with the integer referring to the number of layers in the unit cell. To verify our sample's crystal structure, we performed Raman spectroscopy at 532 and 632.8 nm with x-ray diffraction characterization shown in Supplementary Figure 2. Raman spectra allows easily to distinguish 1T-phase since it has fundamental modes E_{1g} = 292 cm^{-1} and A_{1g} = 402 cm^{-1},[1] whereas their positions E_{1g} = 383 cm^{-1} and A_{1g} = 408 cm^{-1} for 2H- and 3R-states are the same owing to their similar in-plane atoms arrangement.[2] Luckily, Lee and co-workers thoroughly analyzed Raman spectra for these phases.[3] They proved that the presence of 3R-phase in 2H-configuration is accompanied by the significant magnification of intensities for *a*- and *b*-peaks at λ_{exc} = 632.8 nm (see Supplementary Figure 2a-b), which is absent in our case. Thus, it validates 2H-MoS_2 purity of the samples. As an additional verification, we performed x-ray diffraction (XRD) analysis and unambiguously checked the crystal structure because 2H- and 3R-configurations result in different diffraction[4,5] patterns shown in Supplementary Figure 2b. Apart from crystal structure, XRD also provides information about crystallographic parameters, which in our case $a = b = 0.310 \pm 0.005$ nm and $c = 1.229 \pm 0.001$ nm.

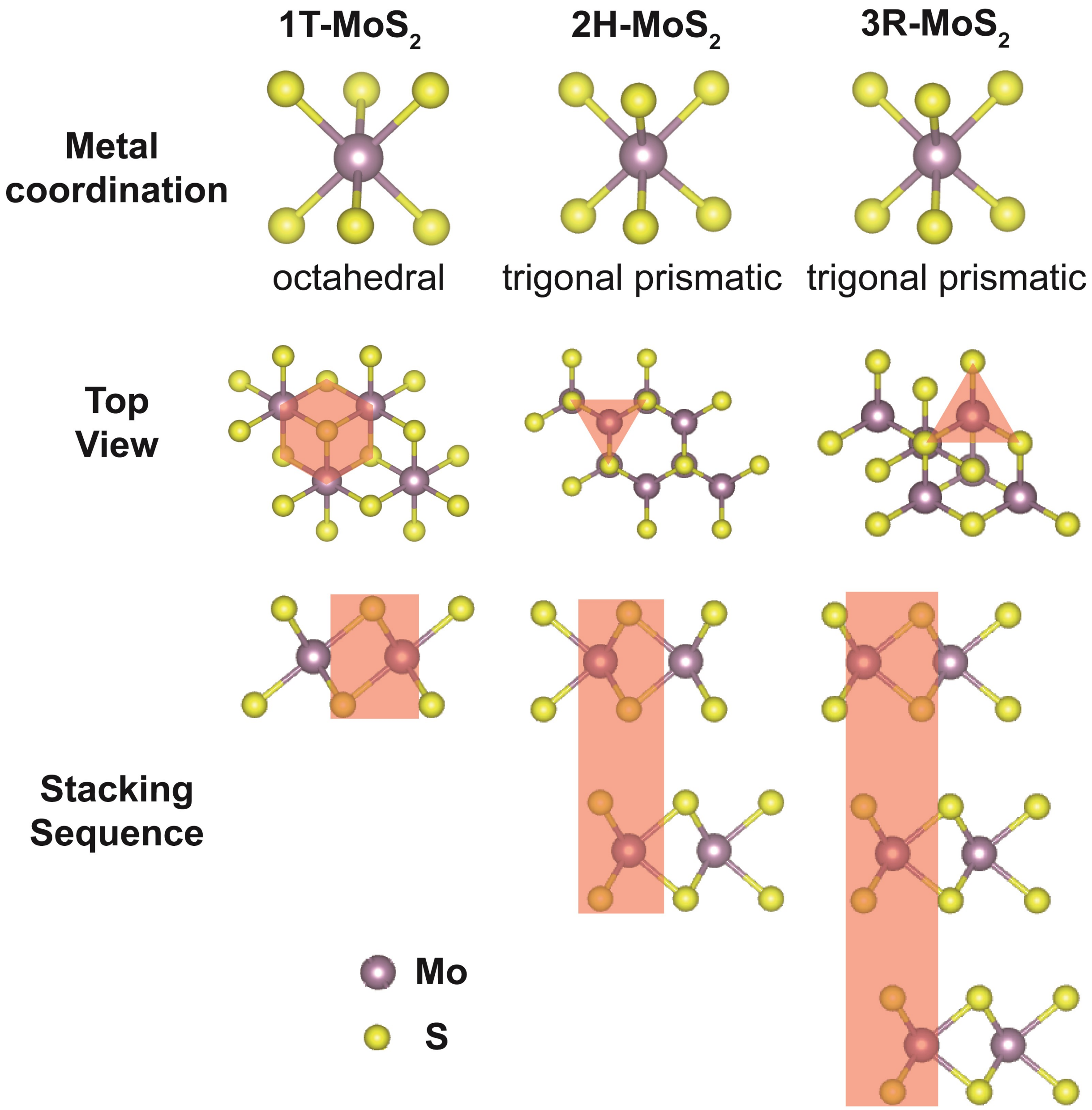

Supplementary Figure 1. Crystal structure of the MoS_2 polytypes: 1T-, 2H-, and 3R-configurations. The red boxes denote the unit cell.

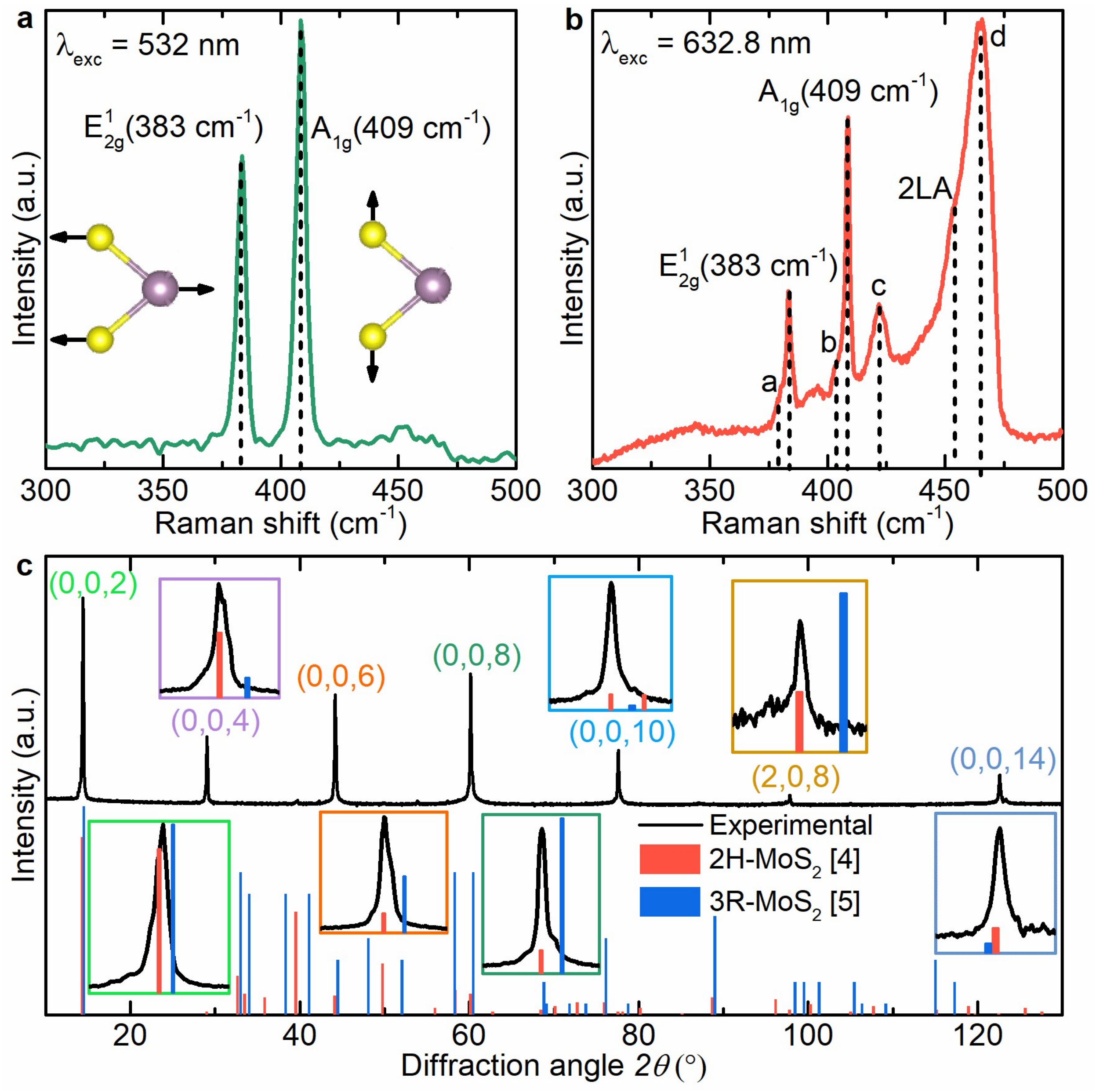

Supplementary Figure 2. Crystal characterization. Raman spectra in **a** non-resonant (excitation wavelength does not induce exciton) and **b** resonant (excitation wavelength induces exciton) conditions. The positions of two first-order Raman modes, namely E_{2g}^{1} and A_{1g}, correspond to 2H-MoS_2. **c** X-ray measurements using Cu-Kα radiation (λ = 0.1542 nm) allows to distinguish 2H- from 3R-configuration[4,5] of MoS_2. The insets in **(c)** shows the magnified diffraction orders.

Supplementary Note 2: Ellipsometry measurements and analysis

We characterize the far-field optical response of MoS_2 through imaging spectroscopic ellipsometry,[6] which configuration is schematically shown in Supplementary Figure 3. The major advantage of imaging over conventional ellipsometry is multiple samples measurements within the same field of view, as illustrated in Figure 1b. As a result, we have multiple spectra in Supplementary Figure 4 for the same system $MoS_2/SiO_2/Si$, but with different thicknesses (104 and 126 nm). Therefore, we model them simultaneously with equal dielectric permittivities. Such an approach gives the most accurate results since it increases data reliability and reduces the correlation between fitting parameters. At the same time, to adequately describe optical constants, it is imperative to obtain a good initial guess for refractive indices for software to find the right answer and a physical model. To overcome the first problem we proceeded in the following way: in the transparent range (800 – 1700 nm) both in-plane and out-of-plane components were described by a Cauchy model $A + B/\lambda^2$, then we performed Kramers-Kronig consistent B-spline expansion[7] for the whole spectral interval (360 – 1700 nm) for in-plane dielectric permittivity. Later we leveraged the Tauc-Lorentz oscillator model to describe in-plane dielectric response by fitting the B-spline result.[8]

Supplementary Equation 1. Tauc-Lorentz oscillator.

$$\varepsilon_2 = \begin{cases} \frac{1}{E} \cdot \frac{A E_0 C (E - E_g)^2}{(E^2 - E_0^2)^2 + C^2 E^2} & for\ E > E_g \\ 0 & for\ E < E_g \end{cases},$$

where E is the photon energy, A is the strength of the oscillator, C is the broadening term, E_g is the optical band gap, and E_0 is the peak central energy with the real part of dielectric function derived from the expression of ε_2 using Kramers-Kronig integration. Finally, we fitted the spectra in Supplementary Figure 4 by varying the parameters of the Tauc-Lorentz oscillators and Cauchy model with the results collected in Supplementary Table 1.

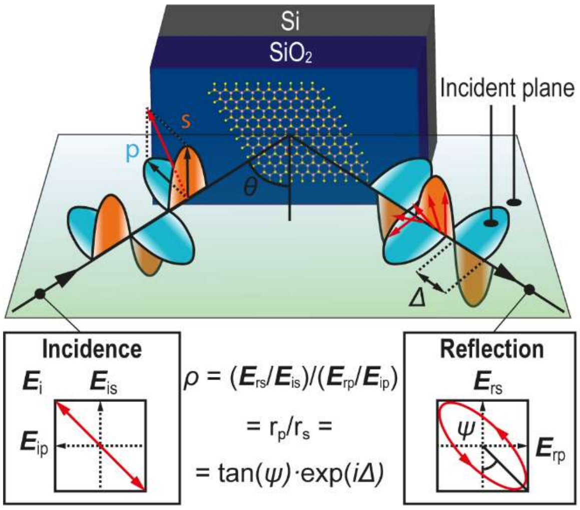

Supplementary Figure 3. The scheme of spectroscopic ellipsometry measurements.

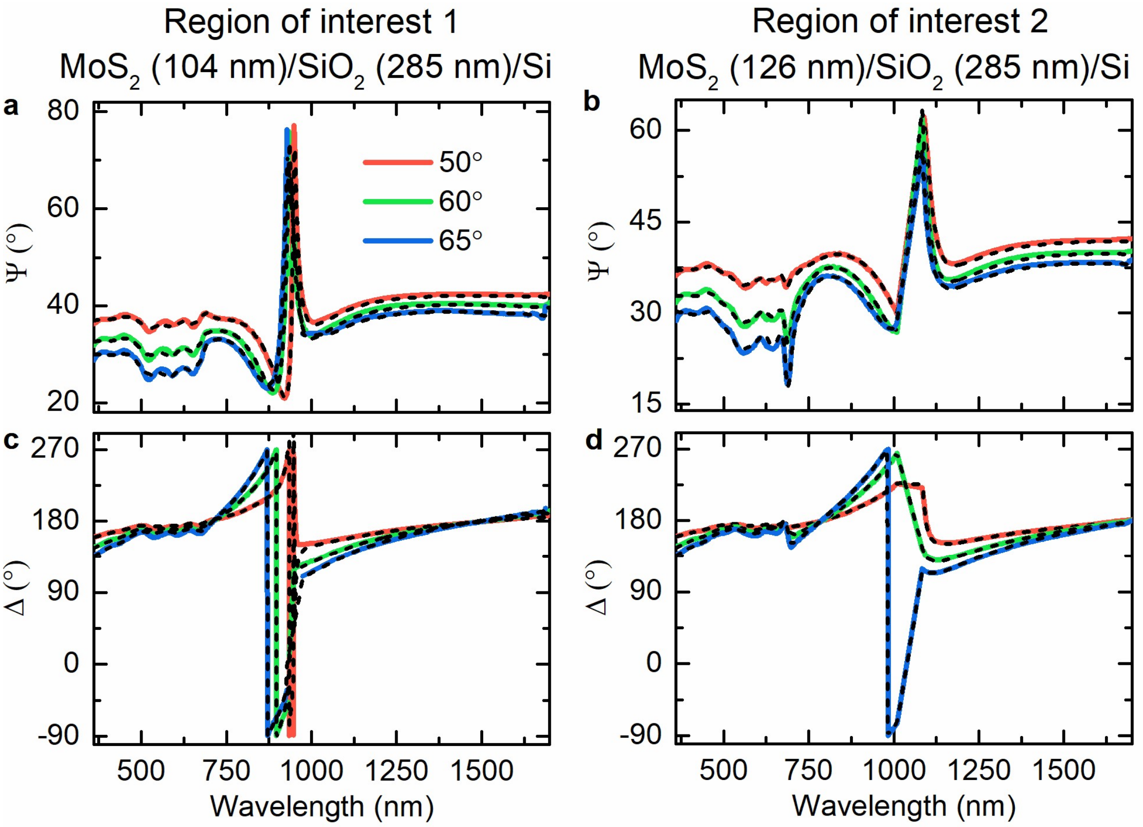

Supplementary Figure 4. a-d Experimentally measured (solid lines) and calculated by Fresnel Formulas[9] (dashed lines) for regions of interest (ROIs) 1 and 2 (shown in Figure 1b) ellipsometric parameters Ψ and Δ for the system $MoS_2/SiO_2/Si$ at three incident angles (50°, 60°, and 65°). The asymmetrical interference-like peak at around 900 nm for ROI 1 and around 1100 nm for ROI 2 is induced by interference enhancement in SiO_2 caused by splitting the incident beam into ordinary

and extraordinary rays. This prominent asymmetry for Ψ stems from giant anisotropy between the *c*-axis and *ab*-plane.

In general, the fitting procedure described above could be summarized in 3 steps, which could be successfully applied to similar TMDCs such as $MoSe_2$, WSe_2, WS_2, and $MoTe_2$ in 2H-configuration. Note that we include in the physical model finite coherence of the light source (bandwidth equals 5 nm for 360 – 1000 nm and 15 nm for 1000 – 1700 nm) to include the device nonidealities:

Step 1 (Transparent fitting region): In the transparent range (800 – 1700 nm), both in-plane and out-of-plane components were described by a Cauchy model $A + B/\lambda^2$. The fitting of Ψ and Δ by Levenberg–Marquardt algorithm results in optical constants presented in Supplementary Figure 5:

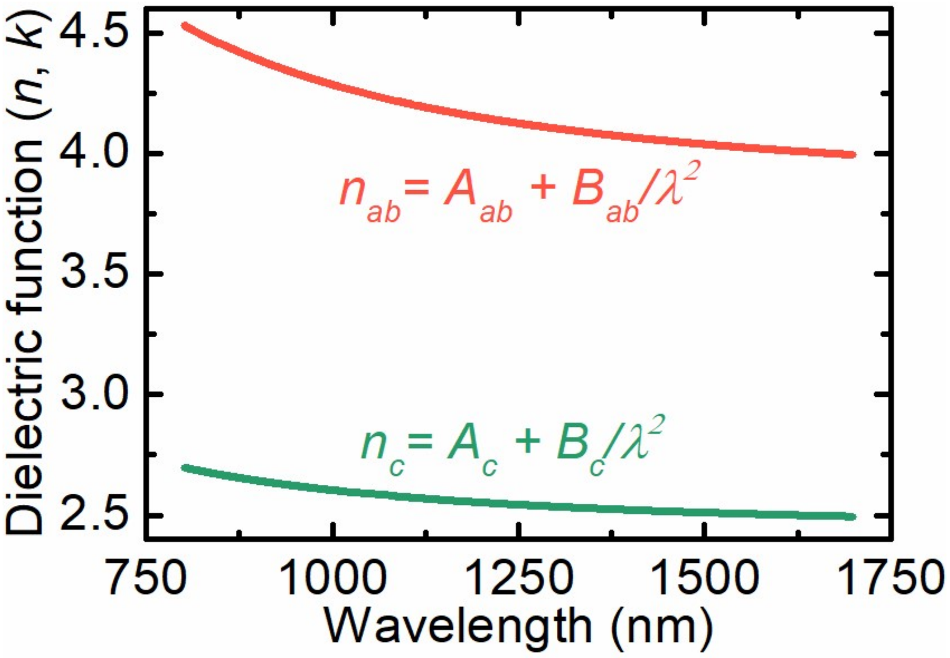

Supplementary Figure 5. Optical constants of MoS_2 along the *ab*-plane and *c*-axis after step 1 with Cauchy parameters $A_{ab} = 3.84$, $B_{ab} = 0.44\ \mu m^2$, $A_c = 2.44$, $B_c = 0.17\ \mu m^2$.

Step 2 (Wavelength expansion): Next, the *ab*-plane dielectric function is approximated by Kramers-Kronig B-splines[7] placed equidistantly in the considered wavelength range with the step

of 0.05 eV, with subsequent expansion of the optical constants fitting to the whole wavelength interval (360 – 1700 nm) by the method described in the work of Mohrmann and et al.[7]:

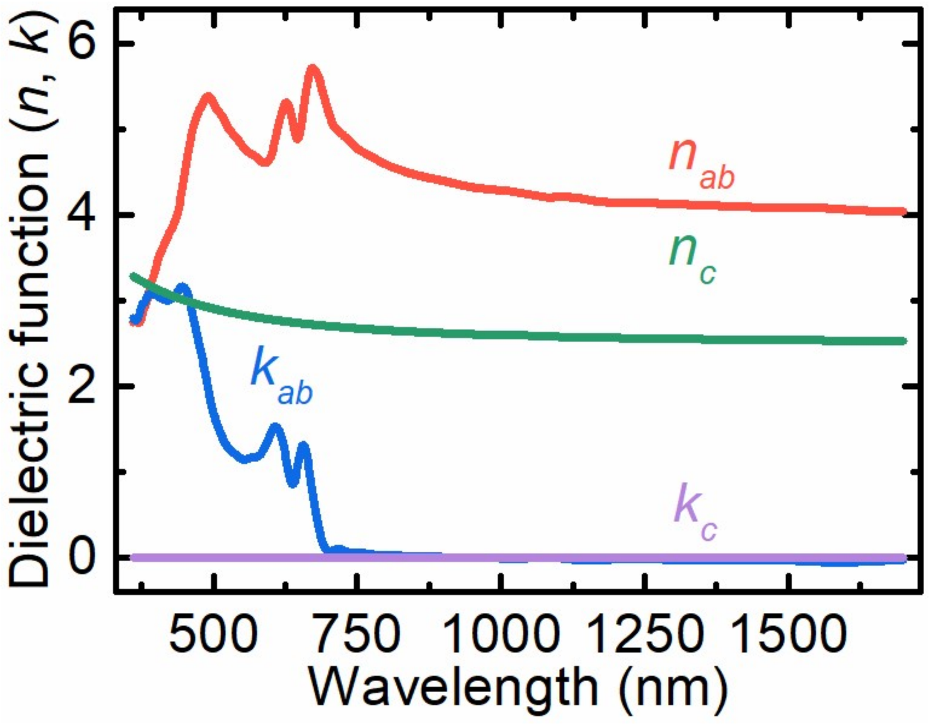

Supplementary Figure 6. Optical constants of MoS_2 along the *ab*-plane (B-splines) and *c*-axis (Cauchy function) after step 2.

<u>Step 3 (Tauc-Lorentz description):</u> Although the previous step already gives decent results, it is worth describing optical constants using the material properties since the B-spline approach tends to provide unphysical optical constants.[7] In the case of TMDCs, the best dielectric function for describing their dielectric function is the Tauc-Lorentz oscillator model.[8] It also allows for obtaining more accurate values (Figure 2a) because of the reduction number of the fitting parameters (from 57 to 21) with the best values collected in Supplementary Table 1.

Supplementary Table 1. Tauc-Lorentz parameters of the oscillators (excitons) describing the in-plane dielectric response of MoS_2 with $\varepsilon_\infty = 5.26 \pm 0.19$ and ultraviolet pole (unbroadened oscillator) placed at 15 eV with amplitude equals to 228 ± 8 that affects the real part of the optical constants by accounting for a strong absorption outside the measured spectrum. In contrast, the

resulting Cauchy model for the out-of-plane component has A = 2.463 ± 0.009 and B = (119 ± 9)·10^3 nm^2.

Oscillator	A eV	E_0 eV	C meV	E_g eV
#1 (A-exciton)	308 ± 6	1.852 ± 0.002	67 ± 5	1.761 ± 0.009
#2 (B-exciton)	135 ± 5	2.006 ± 0.006	148 ± 14	1.82 ± 0.03
#3 (C-exciton)	19.3 ± 0.5	2.662 ± 0.005	380 ± 27	1.24 ± 0.12
#4 (C'-exciton)	69 ± 7	2.99 ± 0.03	1348 ± 39	1.31 ± 0.03

Supplementary Note 3: Interference enhancement for anisotropic optical constants determination

Identification of anisotropic properties of high index semiconductors by traditional techniques such as reflectance, transmission, and ellipsometry is a tedious task owing to the low sensitivity of the signal to the out-of-plane permitting to a determination of only in-plane dielectric response.[10] To overcome this obstacle, we used interference enhancement method. In the approach, the sample of interest is placed on SiO_2/Si wafer. The SiO_2 layer accomplishes two functions. First of all, it increases sensitivity to the p- and s-polarization reflection at the interface MoS_2/SiO_2 due to their large difference in their refractive indices ($n_{ab} \sim 4$ and $n_c \sim 2.5$ for MoS_2 and $n \sim 1.4$ for SiO_2). Secondly, oxide produces interference peaks (at 950 and 1090 nm in Figure S2a-b), which depends on MoS_2 and SiO_2 thicknesses, allowing to implement Step 1 of the fitting procedure because of the major difference between isotropic and anisotropic treatment in the peak vicinity as illustrated in Supplementary Figure 7.

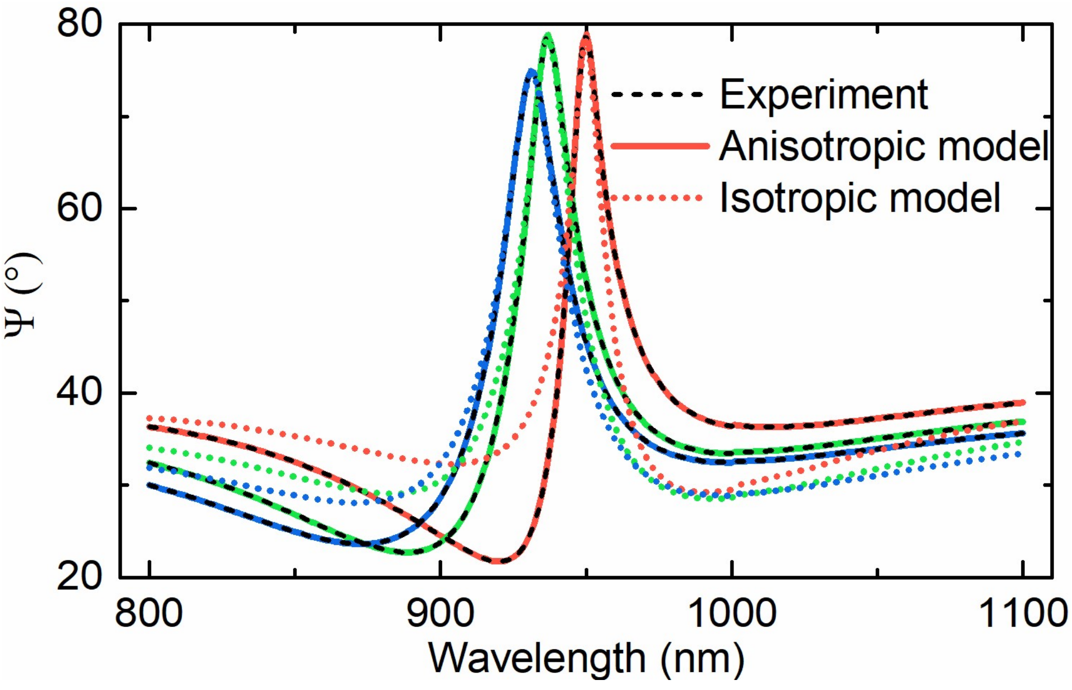

Supplementary Figure 7. Interference peak caused by the SiO_2 layer in MoS_2 (104 nm)/SiO_2 (285 nm)/Si system. The giant anisotropy in MoS_2 transforms almost symmetrical peak into asymmetrical.

Supplementary Note 4: Dielectric permittivity of MoS_2 from first principles

The comparison of the experimental data with first-principle calculations (see Methods) is presented in Supplementary Figure 8. Our experimental results qualitatively and in the infrared region, even quantitatively agree with the first-principles calculations, which further validate our findings. The small mismatch is likely attributed to the approximations used in DFT analysis since near-field measurements reproduced our dielectric function at 1470 – 1570 and 632.8 nm in the main text. Surprisingly, the in-plane component of the dielectric permittivity tensor is better described by the microscopic dielectric function, whereas the out-of-plane component is better described by the macroscopic dielectric function (for the rigorous definitions of micro- and macroscopic dielectric response see the book of Bechstedt[11]). Clearly, our findings indicate that for layered materials, the physical origins for the spectral behavior of the in-plane and out-of-plane components of the dielectric permittivity tensor are different and cannot be treated in the same way. To date, the authors believe that this phenomenon stems from the similarity of *ab*-plane to monolayer structure, where the local effects (microscopic) play a significant role in optical response. Still, further studies are needed to clarify the observed effect, which is fundamental for *ab initio* study of the layered materials.

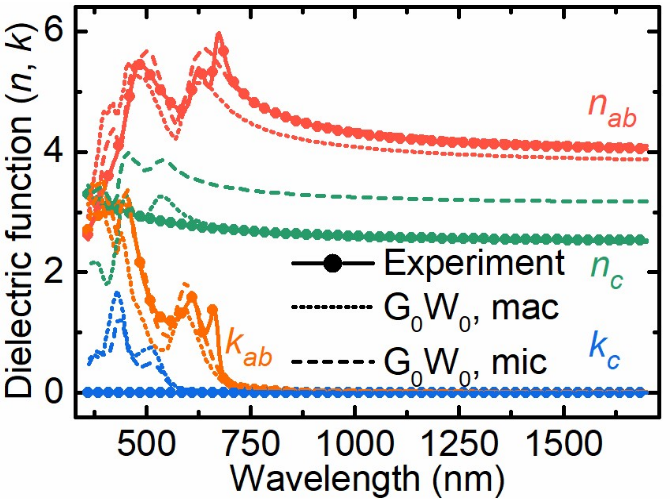

Supplementary Figure 8. Comparison between experimental and DFT predicted macroscopic (mac) and microscopic (mic) optical constants along the crystallographic *ab*-plane and *c*-axis.

Supplementary Note 5: Near-field imaging of a planar waveguide mode within MoS_2 flake and extreme skin depth in silicon

To analyze the planar waveguide modes in MoS_2 flake and silicon waveguide on MoS_2 with extreme skin-depth at multiple wavelengths, we performed scattering-type near-field optical microscopy (s-SNOM), which scheme is presented in Supplementary Figure 9. The resulting signals are an oscillation of light intensity induced by the tip and edge-scattered photons, as clearly seen in Supplementary Figures 10-12. To analyze the effective mode index, we carried out the complex Fourier transformation (FT). Unlike the widely used real FT, it takes into account the real and imaginary signals at the same time and thus more precise, as could be understood from Supplementary Figure 13. Furthermore, it provides additional information about the predominant scattering mechanism. Mainly, there are no peaks for the negative values of q in Supplementary Figures 10-12c, indicating that no modes propagate in the backward direction (from the edge to the tip).

Besides, it is worth to discuss the absence of TM_3, TM_2, and TM_0 modes at λ = 632.8 nm in the measured signal in Supplementary Figure 13a. The first two TM_3 and TM_2 could not propagate because their figure of merit or, in other words, the possible number of observed fringes, is less than unity; while the coupling efficiency f with an s-SNOM tip for TM_0 (f = 0.016) is much lower than for TM_1 (f = 0.043) as demonstrated in Supplementary Figure 14, thus explaining the predominant behavior of TM_1 in the measured signal in Supplementary Figure 11.

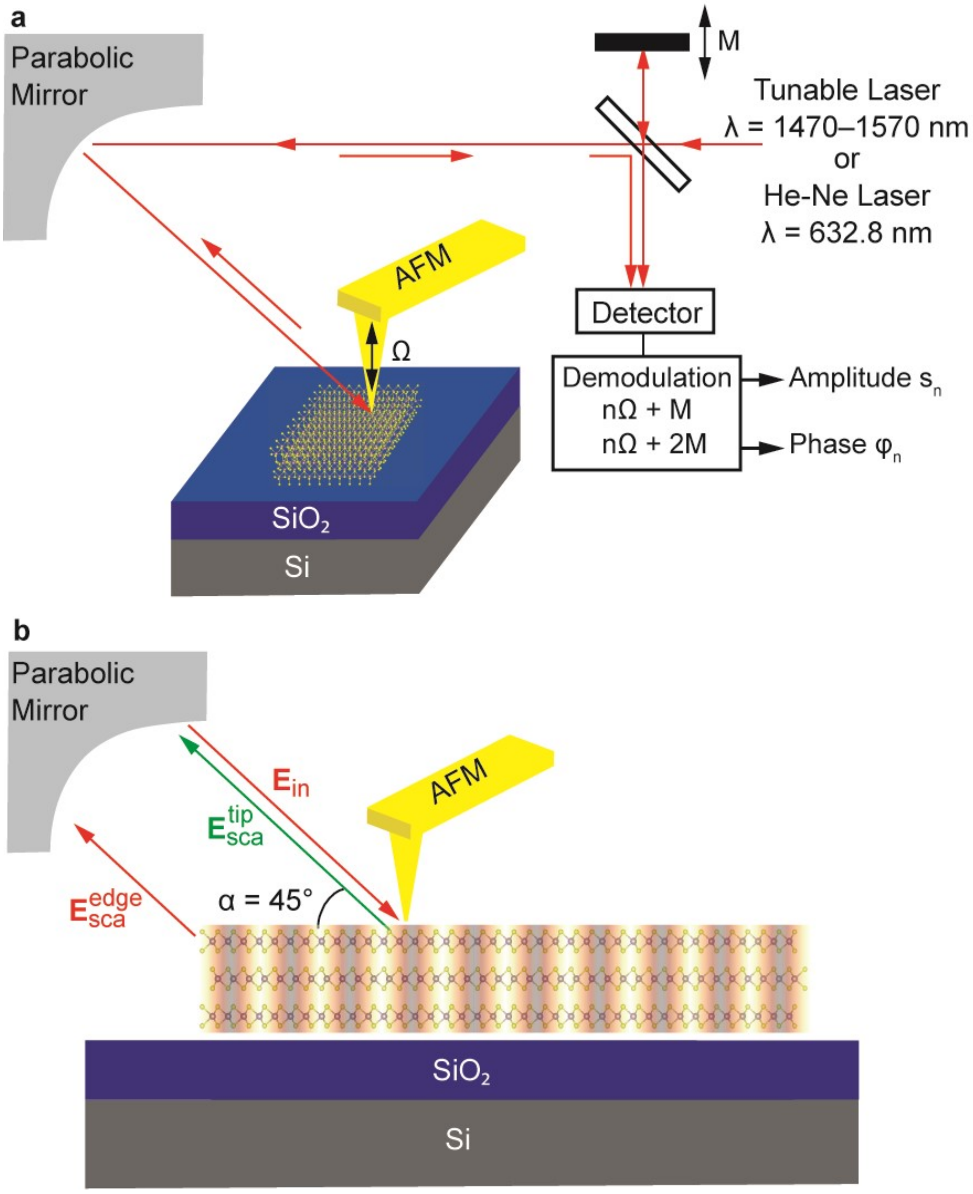

Supplementary Figure 9. a Schematic of the s-SNOM experimental configuration used to image MoS_2 flake. A metalized AFM tip is illuminated by p-polarized light of wavelength λ. It launches a planar waveguide mode, which interferes with the illuminating plane wave and gets scattered at the sample edge to the far-field, where a distant detector collects it. **b** Illustration of waveguide mode tip excitation and following scattering at the flake's edge.

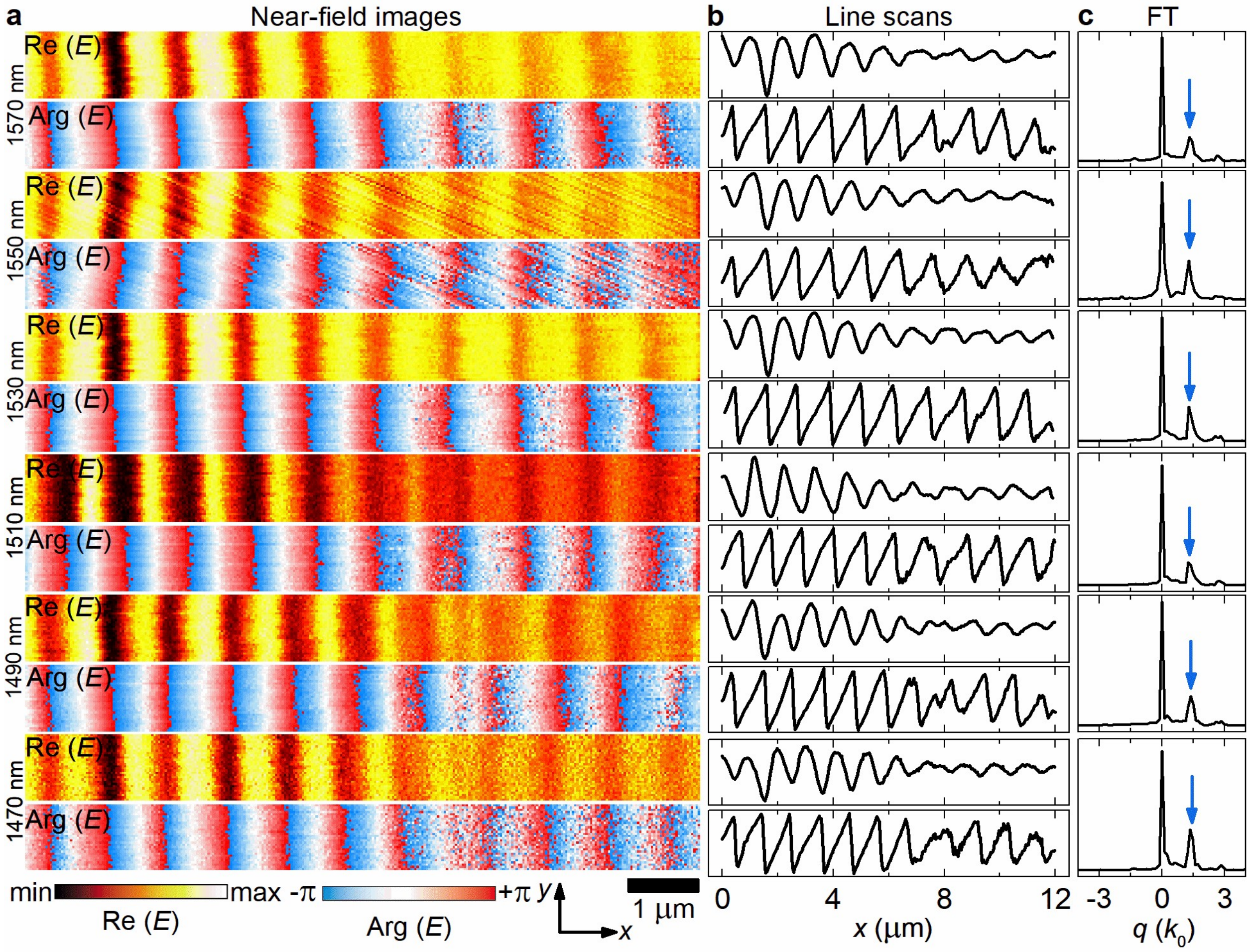

Supplementary Figure 10. a Near-field images, real part Re(E) and phase Arg(E), of electric field E taken at 1570 – 1470 (from top to bottom) in an area of the image in Figure 3b, indicated by a blue rectangular. **b** x-line scans taken from **(a)** and averaged over 1.2 μm along the y-axis. **c** Fourier transform (FT) amplitude of the complex near-field signal in **(b)**, the blue arrow marks the peak associated with waveguide mode.

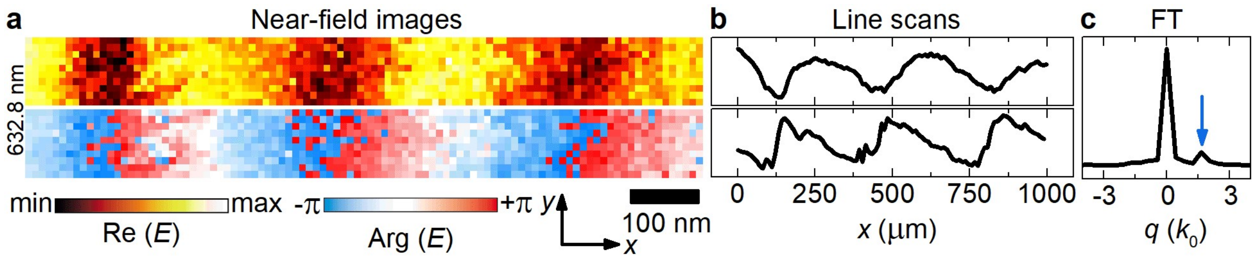

Supplementary Figure 11. a Near-field images, real part Re(E) and phase Arg(E), of electric field E taken at 632.8 nm in an area of the image in Figure 3b, indicated by a blue rectangular. **b** x-line scans taken from **(a)** and averaged over 1.2 μm along the y-axis. **c** Fourier transform (FT)

amplitude of the complex near-field signal in **(b)**, the blue arrow marks the peak associated with waveguide mode.

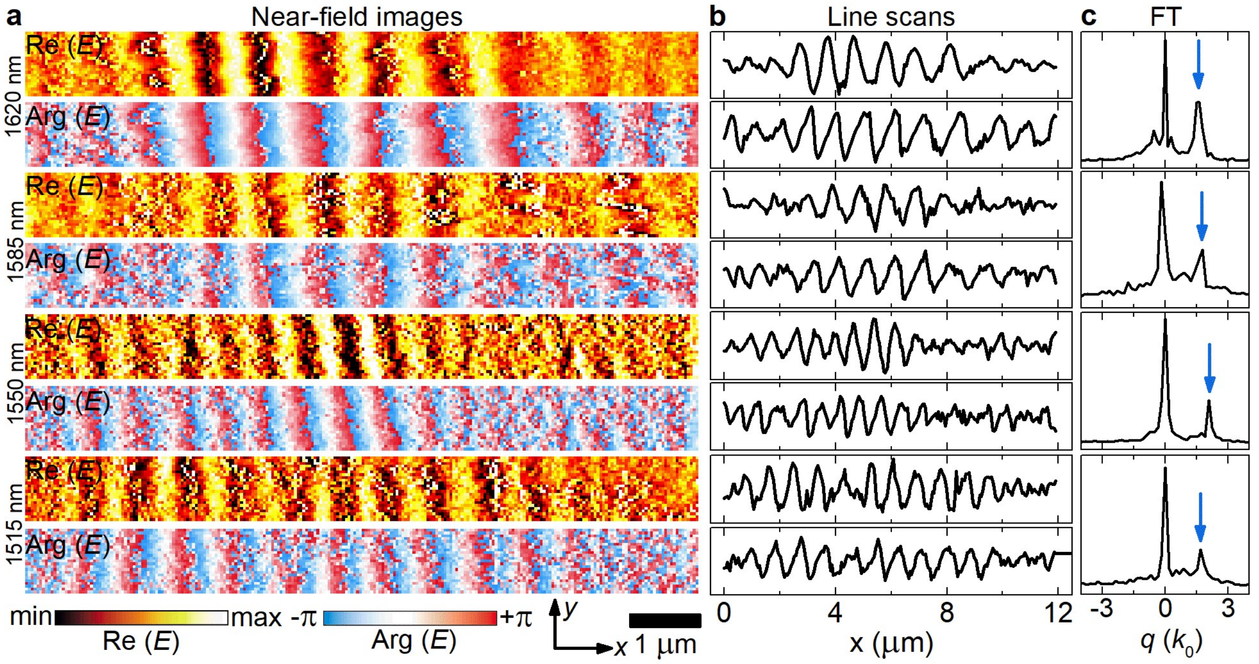

Supplementary Figure 12. a Near-field images, real part Re(E) and phase Arg(E), of electric field E taken at 632.8 nm in an area of the image in Figure 5g-h, indicated by a green rectangular. **b** x-line scans taken from **(a)** and averaged over 1.2 μm along the y-axis. **c** Fourier transform (FT) amplitude of the complex near-field signal in **(b)**, the blue arrow marks the peak associated with waveguide mode.

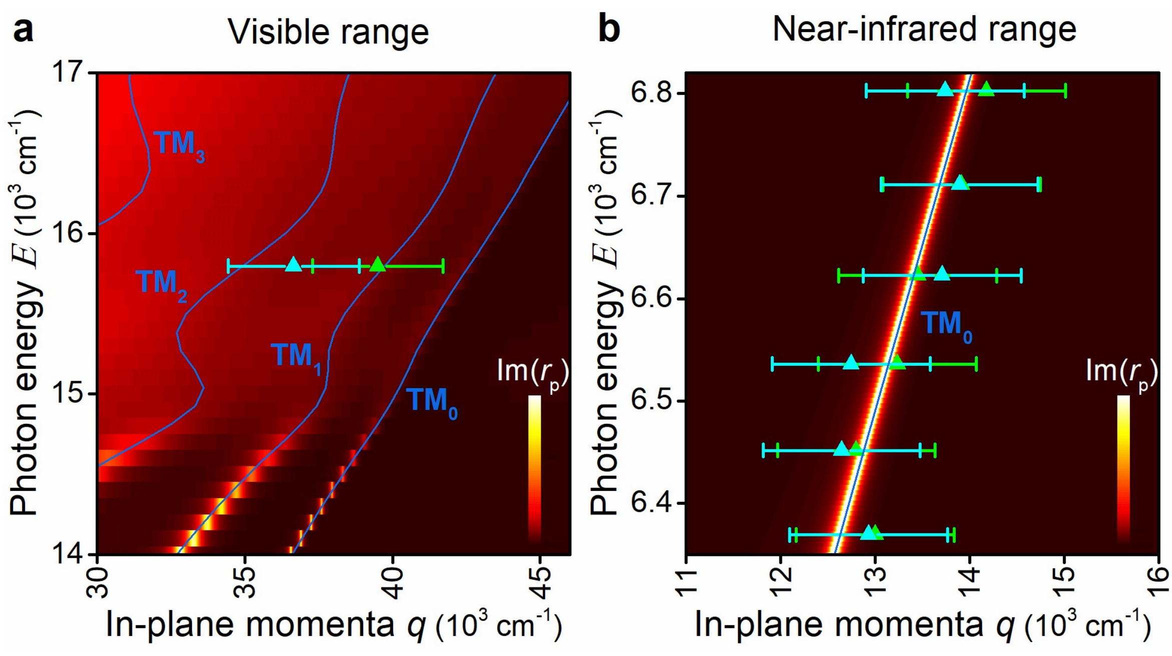

Supplementary Figure 13. Comparison of the different analyses of near-field signal with green and cyan triangles corresponding to complex and real FT, respectively, for **a** visible and **b** near-infrared wavelength ranges. Meanwhile, in the near-infrared interval, different approaches yield the same result; for visible range, the difference is striking with complex FT, giving much better agreement with the theory prediction.

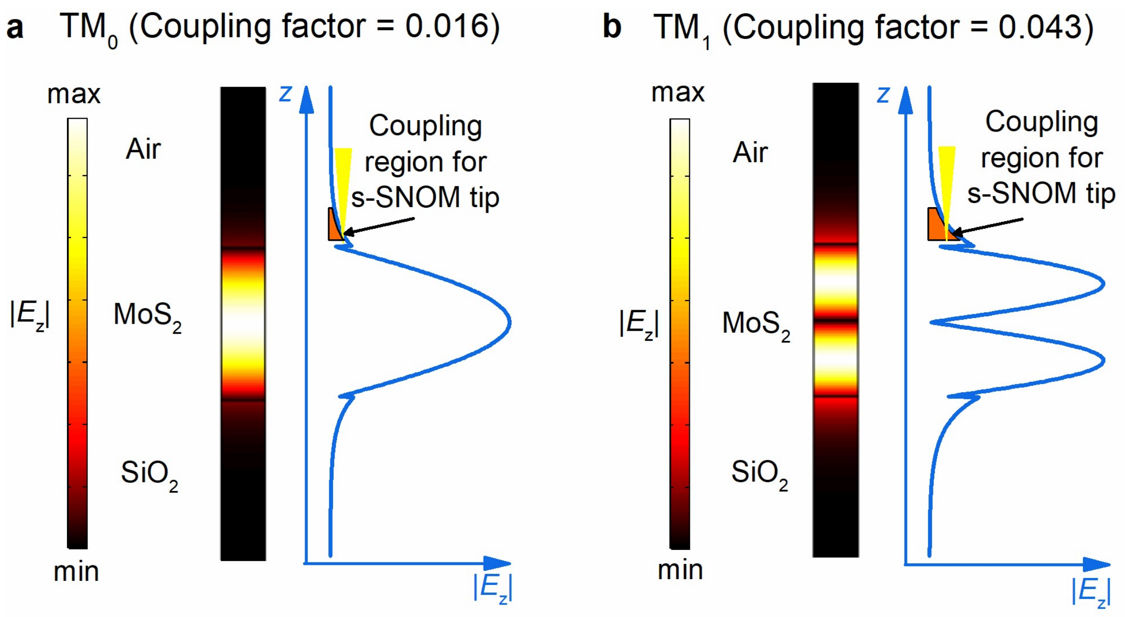

Supplementary Figure 14. Coupling efficiency comparison of waveguide near-field with s-SNOM tip at λ = 632.8 nm for **a** TM_0 and **b** TM_1 modes. Clearly, the coupling factor for TM_1 (0.043) is much higher than for TM_0 (0.016), thus explaining the predominant behavior in the measured signal for TM_1 in Supplementary Figure 13.

SUPPLEMENTARY REFERENCES

1. Fang, Y. *et al.* Structure Re-determination and Superconductivity Observation of Bulk 1T MoS_2 . *Angew. Chemie* **130**, 1246–1249 (2018).

2. Placidi, M. *et al.* Multiwavelength excitation Raman scattering analysis of bulk and two-dimensional MoS_2: Vibrational properties of atomically thin MoS_2 layers. *2D Mater.* **2**, 35006 (2015).

3. Lee, J. U. *et al.* Raman Signatures of Polytypism in Molybdenum Disulfide. *ACS Nano* **10**, 1948–1953 (2016).

4. Mcmurdie, B. H. F. *et al.* Standard X-Ray Diffraction Powder Patterns from The JCPDS Research Associateship — International Centre for Diffraction Data Ammonium Hydrogen Phosphate , $NH_4H_2PO_4$ Barium Magnesium Germanium Oxide ,. *Powder Diffr.* **1**, 334–345

(1986).

5. Takeuchi, W. Nowacki, Detailed crystal structure of rhombohedral MoS_2 and systematic deduction of possible polytypes of molybdenite, *Schweizerische Mineral. Und Petrogr. Mitteilungen* **44**, 105–120 (1964).

6. Funke, S. *et al.* Imaging spectroscopic ellipsometry of MoS2. *J. Phys. Condens. Matter* **28**, 385301 (2016).

7. Mohrmann, J., Tiwald, T. E., Hale, J. S., Hilfiker, J. N. & Martin, A. C. Application of a B-spline model dielectric function to infrared spectroscopic ellipsometry data analysis. *J. Vac. Sci. Technol. B* **38**, 014001 (2020).

8. Ermolaev, G. A., Yakubovsky, D. I., Stebunov, Y. V., Arsenin, A. V. & Volkov, V. S. Spectral ellipsometry of monolayer transition metal dichalcogenides: Analysis of excitonic peaks in dispersion. *J. Vac. Sci. Technol. B* **38**, 014002 (2020).

9. Hecht, E. *Optics* (Pearson, Harlow, 2016).

10. Ermolaev, G. A. *et al.* Broadband optical properties of monolayer and bulk MoS_2. *npj 2D Mater. Appl.* **4**, 1–6 (2020).

11. Bechstedt, F. *Many-Body Approach to Electronic Excitations: Concepts and Applications* (Springer, Berlin, 2014).
